# EZH2 restricts *Tcf7* DNA methylation and promotes T_FH_ differentiation during acute viral infection

**DOI:** 10.3389/fimmu.2022.942465

**Published:** 2022-08-15

**Authors:** Yuan Luo, Dan Li, Luoyingzi Xie, Shun Lei, Xiangyu Chen, Cong Wang, Dong Yao, Lin Li, Jingyi Fang, Cheng Chen, Shijie Yuan, Fei Li, Xiaorong Xie, Yan Zhang, Zhirong Li, Li Hu, Jianfang Tang, Lilin Ye, Zhengping Wei, Ran He

**Affiliations:** ^1^ Department of Immunology, School of Basic Medicine, Tongji Medical College, Huazhong University of Science and Technology, Wuhan, China; ^2^ Institute of Immunology, Third Military Medical University, Chongqing, China; ^3^ Department of Otorhinolaryngology, Union Hospital, Tongji Medical College, Huazhong University of Science and Technology, Wuhan, China; ^4^ Chongqing General Hospital, University of Chinese Academy of Sciences, Chongqing, China; ^5^ Department of Anesthesiology, Chongqing Public Health Medical Center, Chongqing, China; ^6^ Dermatology Hospital, Southern Medical University, Guangzhou, China

**Keywords:** LCMV, T_FH_ cells, EZH2, DNA methylation, *Tcf7*

## Abstract

Follicular helper T (T_FH_) cells provide specialized help for B cells to ensure optimal humoral immunity. The histone methyltransferase EZH2, as a chromatin repressor, secures the T_FH_ differentiation by promoting T_FH_ lineage associated gene expression during acute viral infection, including *Tcf7* and *Bcl6*. By using conditional deletion murine system, we observed that EZH2 ablation in CD4^+^ T cells was accompanied by aberrant accumulation of DNA methyltransferases (DNMTs) DNMT1 and DNMT3B in T_FH_ cells. And the loss of EZH2 promoted aggravation of DNA methylation status at *Tcf7* locus. Therefore, our findings suggested that EZH2 plays an important role in maintenance of hypomethylation at *Tcf7* locus thus affecting T_FH_ differentiation during acute viral infection.

## Introduction

Upon antigen engagement, antigen-specific naive CD4^+^ T cells differentiate into distinct effector populations to execute immune response under the regulation of specific transcription factors (1). Follicular helper T (T_FH_) cells are a subset of CD4^+^ T cells specialized in helping B cells by inducing the formation and maintenance of germinal centers (GCs), which are indispensable for differentiation of high-affinity antibody-producing plasma cells and production of memory B cells (2, 3). Abnormal T_FH_ cell differentiation is closely related to antibody mediated autoimmune diseases, such as systemic lupus erythematosus and rheumatoid arthritis (4–6). Hence, dissecting the differentiation of T_FH_ cells can help to modulate the humoral immunity for better control of infection or alleviation of autoimmune diseases (7).

T_FH_ differentiation is characterized as a multi-stage process, which is precisely regulated by multiple transcription factors. The transcriptional repressor Bcl6 is the “master regulator” of T_FH_ differentiation and is essential for the development of T_FH_ cells (3). Moreover, induced ablation of Bcl6 converts “ex-T_FH_” cells into T_H_1 cells during acute lymphocytic choriomeningitis virus (LCMV-Armstrong) infection, suggesting that Bcl6 is critical for the integrity of T_FH_ cells (8). On the contrary, Blimp1 (encoded by *Prdm1*), as the antagonist of Bcl6, promotes the differentiation of non-T_FH_ effector cells by repressing Bcl6 expression (9, 10). Recent studies have shown that TCF1 (encoded by *Tcf7*) acts as upstream hub of the reciprocal antagonistic Bcl6-Blimp1 axis and secures T_FH_ differentiation program by promoting Bcl6 expression but repressing Blimp1 expression, as manifested by the fact that the deficiency of TCF1 restricts the T_FH_ differentiation and effector function (11, 12). Additionally, TCF1 maintains the transcriptional and metabolic signatures of T_FH_ cells, it is not only necessary for adequate expansion of T_FH_ cells, but also critical for T_FH_ cell responses during LCMV infection (13). Other regulators, such as ICOS, Ascl2, Id2, STAT3, Klf2, and Foxo1 were also identified to regulate T_FH_ differentiation (14–17). In addition to transcription mechanisms, epigenetic modification also plays a vital role in cell differentiation and plasticity by responding rapidly to external stimuli and incorporating a variety signals (18). For instance, the SUV39H1-dependent H3K9me3 is important for lineage integrity of T_H_2 cells ​ (19), and the G9a-mediated H3K9me2 is involved in the control of T_REG_ cell differentiation (20). Although the phenotypic and functional changes that occur during T_FH_ differentiation have been well characterized, the detailed epigenetic mechanisms which control T_FH_ differentiation remains little understood.

Enhancer of zeste homolog 2 (EZH2), the catalytic subunit of Polycomb complex 2 (PRC2), mediates the trimethylation at lysine 27 of histone H3 *via* its methyltransferase activity (HMT) (21). Generally, the EZH2-dependent H3K27me3 modification is associated with gene silencing through chromatin compaction (22). EZH2-mediated H3K27me3 has been demonstrated to restrict the differentiation and cytokine production through occupying *Tbx21* and *Ifng* loci in T_H_1 cells, and the *Gata3* and *Il4* loci in T_H_2 cells (23). In T_REG_ cells, the H3K27me3 deposition is also required for the repressive gene program, thus maintaining the lineage identity after activation (24). Moreover, EZH2 promotes T_FH_ differentiation potentially by stabilizing the chromatin accessibility of T_FH_ lineage associated genes, and the deletion of EZH2 caused reduced expression of T_FH_ associated genes (25, 26). However, EZH2-mediated H3K27me3 deposition has not been observed at T_FH_ lineage associated gene loci (25). Besides, it remains ambiguous whether and how the chromatin repressor EZH2 promotes T_FH_ differentiation by regulating other modifiers that are negatively associated with T_FH_ differentiation program.

In this study, we demonstrated that EZH2 restricts the expression of DNMT1 and DNMT3B, thus may help to maintain the hypomethylation status of *Tcf7* locus. This study illustrated that EZH2 restrains the methylation status at *Tcf7* locus and promotes the differentiation of T_FH_ cells.

## Materials and methods

### Mice and infectious agents

*Ezh2^fl/fl^*, *Cd4*-Cre transgenic mice, and wild type C57BL/6J (CD45.2 and CD45.1) mice were purchased from the Jackson Laboratory. SMARTA (CD45.1, expressing MHC II I-Ab-restricted TCR specific for LCMV glycoprotein amino acids 66–77 epitope) and LCMV-Armstrong strain were generously provided by Dr. Rafi Ahmed (Emory University). 2 × 10^5^ plaque-forming units of LCMV-Armstrong strain with intraperitoneal injection to set up the acute viral infection model in mice. Both sexes were included without randomization or blinding to establish the experiments at the age of 6-10 weeks. All mice were house kept (3-5 mice per cage) in a specific pathogen-free facility with controlled environmental conditions. All experiments were performed according to the guidelines of the Institutional Animal Care and Use Committee of the Third Military Medical University.

### Flow cytometry and antibodies

Single-cell suspensions of spleens from the experimental animals were used for flow cytometry with a FACSCanto II (BD Biosciences). The surface staining was performed in FACS buffer, the anti-CD4 (RM4-5), anti-CD44 (IM7), anti-CD45.1 (A20), anti-CD45R (RA3-6B2) were obtained from Biolegend. For CXCR5 staining, all the surface antibodies were mixed in FACS buffer (PBS with 2% FBS) containing 2% normal mouse serum and 1% BSA. The CXCR5 staining was performed with a three-step protocol: firstly the cells were stained with purified rat anti-CXCR5 (2G8) at 4°C for 1h; then the cells were washed and stained with biotin-conjugated goat anti-rat IgG (Jackson ImmunoResearch) on ice for 30 min; lastly the cells were washed and stained with fluorescently-labeled streptavidin (Biolegend) and other surface antibodies on ice for 30 min. Ezh2 (11/EZH2) was obtained from BD Biosciences, the staining was performed with a Cytofix/Cytoperm Fixation/Permeabilization Kit (554714, BD Biosciences) according to the manufacturer’s instructions after surface staining. TCF1 (C46C7), DNMT1 (D63A6), DNMT3A (D23G1), DNMT3B (E4I4O) were obtained from Cell Signaling Technology, while Blimp1 (5E7), Foxp3 (3G3) were obtained from BD Biosciences. The staining was performed with a Foxp3/Transcription Factor Staining Buffer Set (eBioscience) according to the manufacturer’s instructions after surface staining. All data were analyzed by FlowJo (Treestar).

### Adoptive transfer

A total of 2 × 10^4^ transgenic CD45.1^+^ SMARTA cells were harvested from naive mice and adoptively transferred intravenously to CD45.2^+^ C57BL/6 mice. On the following day, the recipient mice were intravenously infected with 2 × 10^5^ pfu of LCMV-Armstrong strain.

### Cell sorting

The cell sorting was performed on a FACSAria II (BD Biosciences). The Naive SMARTA cells (CD25^-^CD44^-^CD62L^+^CD4^+^) were sorted from naive SMARTA mice, T_FH_ cells (SLAM^-^CXCR5^high^CD4^+^) were sorted from mice infected with LCMV-Armstrong strain on day 4 and day 8 after CD4^+^ T cells enrichment. The biotin-conjugated antibodies: CD8 (53-6.7), CD45.2 (104), B220 (RA3-6B2), CD11b (M1/70), CD11c (N418), TER119 (TER), NK1.1 (PK136), F4/80 (BM8), CD25 (PC61) were used for the T cells enrichment. The purity of the sorted cells was >95% in all experiments.

### Quantitation of mRNA levels by RT-PCR

For comparison the gene expression of target genes, total RNA was isolated from the cells sorted from mice infected with LCMV Armstrong strain. RNA was extracted with the RNeasy Mini Kit (74104, Qiagen) and reverse transcribed with the RevertAid H Minus First Strand cDNA Synthesis Kit (K1632, Thermo Scientific). Quantitative real-time PCR of target transcripts with appropriate primers (**Supplementary Table 1**) were carried out with SYBR Green PCR kit (208054, Qiagen) on a CFX96 Touch Real-Time System (Bio-Rad). Fold differences in expression levels were calculated according to the 2^−ΔΔCT^ method.

### Western blot analysis

The transfected 293T cells were washed with ice-cold PBS twice, and then lysed in RIPA buffer. Equal amounts of protein from each sample were separated with 10% SDS-PAGE and then transferred to PVDF membranes (IPVH00010, Millipore). The membranes were blocked with 5% bovine serum albumin (B2064, Sigma) for 1 hour at room temperature, and then incubated with appropriate antibodies overnight at 4°C. After four times washing, the membranes were incubated with horseradish peroxidase-conjugated secondary antibody for another one hour at room temperature. Immunoblots were visualized with SuperSignal^®^ West Pico Chemiluminescent Substrate (34080, Thermo Scientific) on a Bio-Rad XRS chemiluminescence detection system (Bio-Rad).

### Protein stability and immunoprecipitation

The full-length cDNA of *Ezh2* was subcloned into pcDNA3.1-HA vector and *Dnmt1*, *Dnmt3a*, *Dnmt3b* were subcloned into pcDNA3.1-FLAG vector for expression. For protein stability studies, the pcDNA3.1-EZH2-HA vector was co-transfected with pcDNA3.1-FLAG-DNMT1 and pcDNA3.1-FLAG-DNMT3B vectors into 293T cells by using TranIT-293 Transfection Reagent (MIR 2705, Mirus), respectively. After 24 h, the transfected cells were treated with cycloheximide for 24 h, and the working solution of cycloheximide was 200 μg/mL. For immunoprecipitation studies, the 293T cells were transfected with pcDNA3.1-FLAG-DNMT1, pcDNA3.1-FLAG-DNMT3A, pcDNA3.1-FLAG-DNMT3B, pcDNA3.1-FLAG vectors. Cells were harvested 48 h after transfection, the extracted product were incubated with 2 μg of anti-Ezh2 (D2C9, Cell Signaling Technology), 2 μg of anti-FLAG (M2; Sigma-Aldrich) for 5 h, and then incubated with Dynabeads Protein G (10004D; Life Technologies) for 2 h. After washing, the protein stability and immunoprecipitated samples were analyzed by immunoblot analysis with appropriate antibodies.

### Genomic methylation by bisulfite sequencing

The bisulfite sequencing of the target genomic region was used to measure the allelic frequency of methylated cytosines. Genomic DNA from the sorted cells were extracted with QIAamp^®^ DNA Mini Kit (51304, Qiagen), and then bisulfate modified by EpiTect^®^ Bisulfite Kit (59104, Qiagen). The bisulfite-modified DNA was amplified with locus-specific primers (**Supplementary Table 1**). The amplified product was cloned into the pMD19-T TA cloning Vector (6013, Takara), then transformed into DH5α competent cells. Independent colonies were sequenced to determine the methylation status from each sample. The CpG island was defined, and primer was designed by website of Li Lab (http://www.urogene.org/methprimer/) (27).

### Statistical analysis

Statistical analysis was performed using Prism version 6.0 (GraphPad) software. Statistical significance was determined by unpaired two-tailed Student’s *t* test. A *P* value of less than 0.05 was considered statistically significant.

## Results

### Epigenetic regulator EZH2 controls T_FH_ differentiation

**Figure 1:**
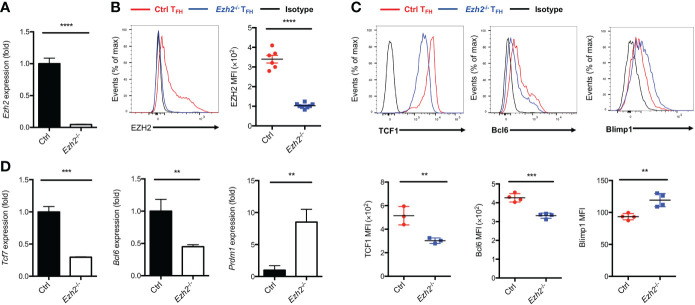
Epigenetic regulator EZH2 controls T_FH_ differentiation. The control (*Ezh2* ^fl/fl^) and *Ezh2* ^fl/fl^ *Cd4*-cre (*Ezh2* ^-/-^) mice were infected with 2 × 10^5^ plaque-forming units LCMV-Armstrong strain. After 8 days of infection, T_FH_ (SLAM^-^CXCR5^hi^CD44^+^), sorted to perform the following experiments. **(A)** Analysis of mRNA of *Ezh2* in naive splenic CD4^+^ T cells from control and *Ezh2* ^-/-^ mice *via* RT-PCR. Normalized to their expression in control CD4^+^ T cells. **(B)** Measurement of EZH2 expression in T_FH_ cells *via* flow cytometry from control and *Ezh2* ^-/-^ mice. **(C, D)** Measurement of TCF1, Bcl6 and Blimp1 expression *via* flow cytometry, and the expression of *Tcf7*, *Bcl6* and *Prdm1* transcripts by RT-PCR in T_FH_ cells from control and *Ezh2* ^-/-^ mice. Normalized to their expression in control T_FH_ cells. *P* value was calculated by unpaired two-tailed Student’s *t* test from triplicate experiments. Error bars indicate mean ± SEM, **P < 0.01, ***P < 0.001, ****P < 0.0001.

EZH2 is a subunit of PRC2, which acts as “writer” of the repressive H3K27me3 modification (21). We set out to elucidate the epigenetic role of EZH2 in T_FH_ cells and identify the downstream regulators modulated by EZH2 during acute viral infection. To this end, we crossed mice containing *lox*P-flanked *Ezh2* alleles with mice expressing Cre-recombinase under the control of *Cd4* promoter, enhancer and silencer to generate the conditional knockout mice (*Ezh2* ^fl/fl^ *Cd4*-cre, hereafter called *Ezh2* ^-/-^). The loss of *Ezh2* mRNA in CD4^+^ T cells from *Ezh2* ^-/-^ mice was confirmed by quantitative RT-PCR (**Figure 1A**). We then infected the control (*Ezh2* ^fl/fl^) mice and *Ezh2* ^-/-^ mice with LCMV-Armstrong strain, the deletion of EZH2 protein was validated in T_FH_ cells from *Ezh2* ^-/-^ mice at day 8 after infection (**Figure 1B**). Moreover, the frequency and absolute number of T_FH_ cells in *Ezh2* ^-/-^ mice were significantly diminished compared with those in the control mice (**Supplemental Figure 1A, B**), consistent with previous findings (25, 26). The transcript and protein levels of *Tcf7* and *Bcl6* were both decreased in T_FH_ cells from *Ezh2* ^-/-^ mice, whereas that of *Prdm1* and Blimp1 were increased (**Figures 1C, D**). These results suggested that EZH2 controls the expression of T_FH_ lineage related regulators to secure T_FH_ differentiation during acute viral infection.

### EZH2 regulates the expression of DNA methyltransferases in T_FH_ cells

**Figure 2:**
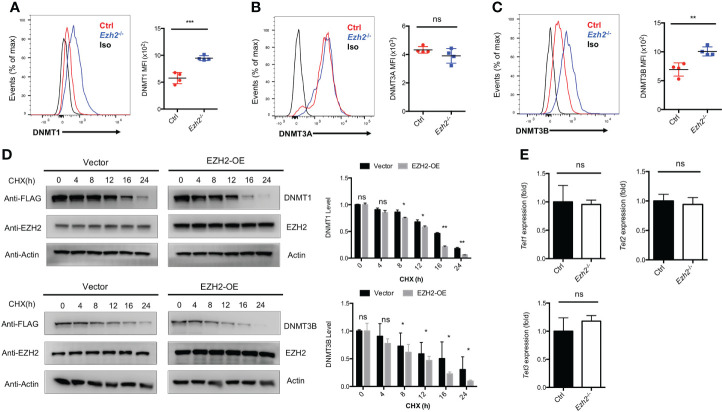
EZH2 regulates the expression of DNA Methyltransferases in T_FH_ cells. **(A–C)** Analysis and summary of the expression of DNMT1, DNMT3A, DNMT3B in T_FH_ cells *via* flow cytometry from control and *Ezh2* ^-/-^ mice at day 8 after infection, respectively. **(D)** The control and EZH2 overexpression-transfected 293T cells were treated with cycloheximide (CHX) at indicated times, followed by western blot analysis. Normalized the expression to the zero timepoint. **(E)** Real-Time PCR analysis of *Tet1*, *Tet2*, and *Tet3* transcripts of T_FH_ cells sorted from infected control and *Ezh2* ^-/-^ mice. Normalized to their expression in control T_FH_ cells. *P* value was calculated by unpaired two-tailed Student’s *t* test from triplicate experiments. Error bars indicate mean ± SEM, ns not significant, *P < 0.05, **P < 0.01, ***P < 0.001.

The transcripts of *Tcf7* and *Bcl6* were decreased with EZH2 deletion in T_FH_ cells, while no deposition of H3K27me3 marks were observed at those loci (25). Thus, EZH2 may regulate other chromatin modifiers to regulate the expression of those genes. It has been reported that EZH2 is involved in DNA methylation pathway to mediate gene expression through interacting with DNMT1, DNMT3A and DNMT3B (28). To determine whether the DNA methylation pathway in T_FH_ cells is affected by EZH2 deletion, we measured the expression of DNA methyltransferases in T_FH_ cells derived from control and *Ezh2* ^-/-^ mice on day 8 after acute viral infection. The DNMT1 and DNMT3B expression were increased with ablation of EZH2 in T_FH_ cells, while the expression of DNMT3A was comparable between EZH2-intact and EZH2-deficient T_FH_ cells (**Figures 2A**–**C**). When the protein synthesis inhibitor cycloheximide was applied to treat the transfected cells *in vitro*, the degradation of DNMT1 and DNMT3B were accelerated by forced expression of EZH2 (**Figure 2D**). These findings implied that DNMT1 and DNMT3B were regulated by EZH2-dependent signaling pathway. Moreover, the transcript level of demethyltransferases *Tet1*, *Tet2*, and *Tet3* were not affected by the ablation of EZH2 in T_FH_ cells (**Figure 2E**).

These data indicated that EZH2 is associated with the DNA methylation pathway but not the DNA demethylation in T_FH_ cells.

### EZH2 deletion elevates the DNA methylation level at the *Tcf7* locus

**Figure 3:**
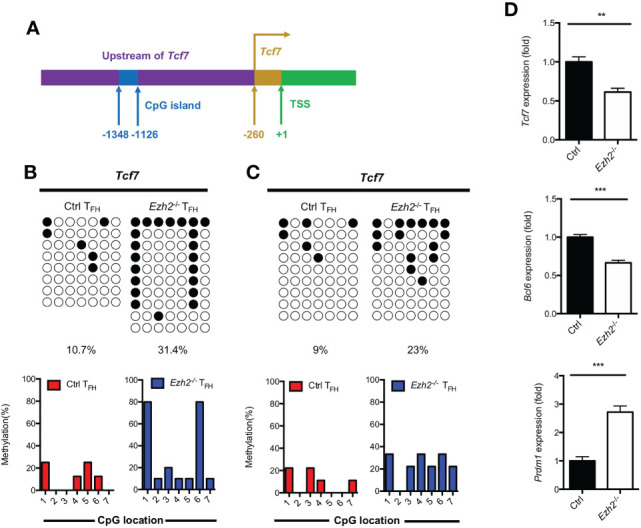
EZH2 deletion elevates the DNA methylation level at the *Tcf7* locus. **(A)** Schematic diagram of CpG island in *Tcf7* gene promoter. **(B)** Bisulfite sequencing analysis and graphical summary of conversed CpG island of *Tcf7* promoter from sorted T_FH_ cells from control and *Ezh2* ^-/-^ mice at day 8 after infection. **(C)** Bisulfite sequencing analysis and graphical summary of CpG island of *Tcf7* promoter from sorted T_FH_ cells from control and *Ezh2* ^-/-^ mice at day 4 after infection. **(D)** Real-Time PCR analysis of *Tcf7*, *Bcl6*, *Prdm1* mRNA of T_FH_ cells sorted from control and *Ezh2* ^-/-^ mice at day 4 after infection. Normalized to their expression in control T_FH_ cells. The horizonal lines were corresponding to the colonies selected for sequencing. Filled black circles indicate methylated cytosine, open white circles indicate nonmethylated cytosine. *P* value was calculated by unpaired two-tailed Student’s *t* test. Error bars indicate mean ± SEM, **P < 0.01, ***P < 0.001.

DNA methylation degree is inversely correlated with the expression of lineage-specific genes during T helper cell development (29, 30). As we know, TCF1 is intrinsically required for T_FH_ cell differentiation (13). To determine whether the increased expression of DNMT1 and DNMT3B were associated with the impaired T_FH_ differentiation, we measured the methylation status at CpG island of key regulator *Tcf7* by bisulfite sequencing. Genomic DNA was isolated from T_FH_ cells sorted from the spleen of control and *Ezh2* ^-/-^ mice at day 8 after infection. Strikingly, the methylation degree of CpG sites in the promoter of *Tcf7* in *Ezh2* ^-/-^ T_FH_ cells was 31.4%, which was three times higher than that of 10.7% in T_FH_ cells from control mice (**Figures 3A, B**; **Supplemental Figure 2A**). Meanwhile, all the CpG sites became methylated in T_FH_ cells from *Ezh2* ^-/-^ mice, and the *Ezh2* ^-/-^ T_FH_ cells were 80% methylated at CpG site 1 and 6, while 3 of 7 CpG sites maintained unmethylated in the control T_FH_ cells (**Figure 3B**). Moreover, the methylation status at *Tcf7* locus was 23% in T_FH_ cells from *Ezh2* ^-/-^ mice at early stage, which was twice higher as that of 9% in T_FH_ cells from control mice (**Figure 3C**; **Supplemental Figure 2B**). Meanwhile, more than 85% of CpG sites became methylated in *Ezh2* ^-/-^ T_FH_ cells, but the control T_FH_ cells maintained 3 of 7 CpG sites unmethylated (**Figure 3C**). Additionally, the *Tcf7* transcripts were diminished in early *Ezh2* ^-/-^ T_FH_ cells, and thus accompanied by decreased expression of *Bcl6* and increased expression of *Prdm1* (**Figure 3D**).

Taken together, these results supported that EZH2 ensured the DNA hypomethylation degree at *Tcf7* locus.

### EZH2 displays inability to affect the methylation status at other gene loci

**Figure 4:**
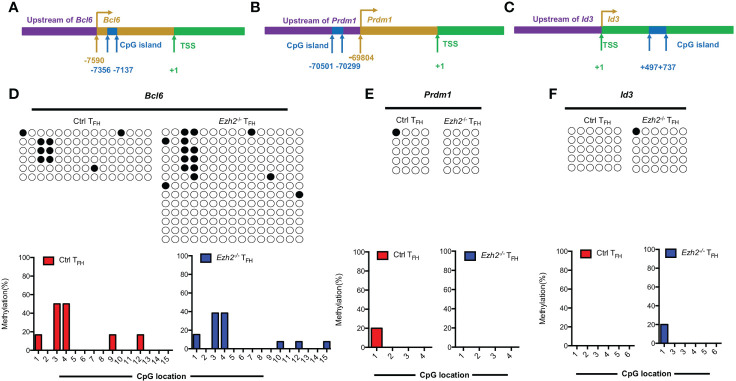
EZH2 displays inability to affect the methylation status at other gene loci. Genomic DNA was extracted from sorted T_FH_ cells derived from control and *Ezh2* ^-/-^ mice at day 8 after LCMV Armstrong strain infection. **(A–C)** Schematic diagram of CpG island at *Bcl6* locus **(A)**, *Prdm1* locus **(B)** and *Id3* locus **(C)**. **(D–F)** The DNA methylation status at *Bcl6* locus **(D)**, *Prdm1* locus **(E)** and *Id3* locus **(F)** were performed by bisulfite sequencing analysis with or without EZH2 ablation. The horizonal lines were corresponding to the colonies selected for sequencing. Filled black circles indicate methylated cytosine, open white circles indicate nonmethylated cytosine.

In addition to the *Tcf7* locus, we also measured the methylation status of CpG islands at other T_FH_ lineage related gene loci from both control and *Ezh2* ^-/-^ T_FH_ cells at day 8 after infection. Bcl6 and Blimp1 are both downstream mediators of TCF1 (11, 12). The methylation status at *Bcl6* locus were demethylated in T_FH_ cells from both control mice and *Ezh2* ^-/-^ mice (**Figures 4A, D**), though its transcripts declined in T_FH_ cells with EZH2 deletion (**Figure 1D**). Meanwhile, the CpG sites in the promoter region of *Prdm1*, were completely unmethylated in T_FH_ cells from both control and *Ezh2* ^-/-^ mice (**Figures 4B, E**), but the expression of *Prdm1* were much higher in T_FH_ cells from *Ezh2* ^-/-^ mice than that from the control mice (**Figure 1D**). In addition, the CpG island of *Id3*, which is another T_FH_ lineage associated factor (16), remained unmethylated in T_FH_ cells from EZH2-intact and EZH2-deficient mice (**Figures 4C, F**).

Taken together, these results together suggested that EZH2 displays inability to regulate the methylation status at *Bcl6*, *Prdm1* and *Id3* loci.

### Methylation of *Tcf7* Locus is associated with the T_FH_ differentiation during acute viral infection

**Figure 5:**
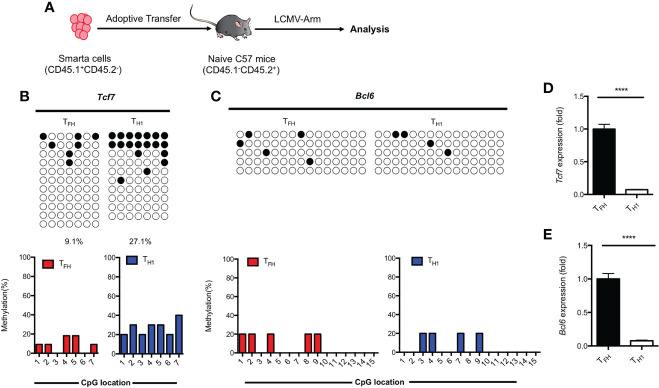
Methylation of *Tcf7* Locus is associated with the differentiation of T_FH_ cells during acute viral infection. **(A)** Procedure of experiment. 2 × 10^4^ naive SMARTA cells were adoptively transferred into naive C57BL/6 mice, the chimeras were infected with LCMV Armstrong strain subsequently. **(B, C)** The virus experienced T_FH_ (CD45.1^+^SLAM^low^CXCR5^+^) and T_H_1 (CD45.1^+^SLAM^high^CXCR5^-^) cells were sorted at day 7 after infection with LCMV Armstrong strain. The isolated genomic DNA purified from these two subtypes were subcloned into pMD19-T TA vectors for subsequent sequencing. The graphical summary of CpG islands of *Tcf7* **(B)** and *Bcl6* **(C)** were performed with bisulfite sequencing. **(D, E)** The transcriptional expression of *Tcf7* **(D)** and *Bcl6* **(E)** were measured by RT-PCR. Normalized to their expression in T_FH_ cells. The horizonal lines were corresponding to the colonies selected for sequencing. Filled black circles indicate methylated cytosine, open white circles indicate nonmethylated cytosine. *P* value was calculated by unpaired two-tailed Student’s *t* test. Error bars indicate mean ± SEM. ****P < 0.0001.

Results obtained from primary T_FH_ cells demonstrated that the regulation of TCF1 expression is associated with methylation status of CpG sites in the promoter region of *Tcf7* (**Figure 3**). To determine whether the methylation status at *Tcf7* locus is associated with antigen specific T_FH_ differentiation during acute viral infection, we transferred naive SMARTA cells into naive recipient mice, then the chimeras were subsequently infected with LCMV Armstrong strain (**Figure 5A**). At day 7 after infection, the CpG sites in *Tcf7* promoter region was 27.1% methylated in antigen specific T_H_1 cells, which was nearly three times higher than that of 9.1% in antigen specific T_FH_ cells (**Figure 5B**, Supplemental **Figure 3A**). And the expression of *Tcf7* and TCF1 were remarkably higher in T_FH_ cells compared with those in T_H_1 cells (**Figure 5D**, Supplemental **Figure 3B**). These results indicated that the methylation status at *Tcf7* locus was inversely correlated with the transcripts and protein expression of *Tcf7*. Meanwhile, the *Bcl6* locus maintained demethylated in T_FH_ cells and T_H_1 cells (**Figure 5C**), while the transcripts and protein of *Bcl6* were much higher in T_FH_ cells compared with those in T_H_1 cells (**Figure 5E**, Supplemental **Figure 3C**).

These findings together suggested that hypomethylation of *Tcf7* locus was positively correlated with the T_FH_ differentiation during acute viral infection.

## Discussion

Besides transcription factors, epigenetic regulators have been demonstrated to involve in regulating T_FH_ differentiation program recently (4, 25, 26, 31, 32). For instance, ablation of EZH2 led to less chromatin accessibility of T_FH_ lineage associated genes (25), which reminded a unique function of EZH2 in transcriptional activation of T_FH_ differentiation program (25). Moreover, EZH2 was associated with H3K27ac rather than H3K27me3 in T_FH_ cells (26). EZH2 thus positively regulates T_FH_ differentiation, despite generally being considered an epigenetic repressor *per se*. Our study further elucidates that EZH2 regulates the epigenetic modification of *Tcf7* and promotes the differentiation of T_FH_ cells. In T_FH_ cells, EZH2 repressed the expression of DNMT1 and DNMT3B, which were associated with the methylation status at *Tcf7* locus in T_FH_ cells. Our study therefore uncovered a specific role for EZH2 in T_FH_ differentiation from an epigenetic perspective and shed a new light on the mechanism of epigenetic modification associated with T_FH_ differentiation program.

In this study, we provided the first evidence that ablation of EZH2 significantly increased the expression of DNMT1 and DNMT3B in T_FH_ cells. We also observed that EZH2 could interact with DNMTs through direct protein-protein interaction (**Supplemental Figure 4**), which was consistent with a previous study (28). The H3K4me3 modification regulator histone methyltransferase SET7, was reported to trigger the degradation of DNMT1 by direct interaction (33), likely through the ubiquitin-proteasome system as shown by direct binding between EZH2 and the E3 ligase NP95 (34). Thus, it is plausible that EZH2 may similarly drive the degradation of DNMT1 and DNMT3B through direct interaction. It will be of great interest to verify the proper mechanism of how EZH2 mediates the expression of DNMT1 and DNMT3B in future studies.

DNA methylation is critical for the regulation of gene expression. We hence focused on the methylation status at T_FH_ lineage related gene loci, including *Tcf7*, *Bcl6*, *Id3* and *Prdm1*. The *Tcf7* promoter was demonstrated as a target locus of DNMT3A, and the expression of DNMT3A was necessary to maintain the methylation status at *Tcf7* promoter to restrict the frequency of memory precursor cells during acute viral infection (35). In this study, we found that *Tcf7* locus was more selectively methylated in T_FH_ cells with EZH2 deletion compared to other key loci were not affected. The inhibited transcriptional activity of *Tcf7* led to less expression of *Bcl6* and more *Prdm1* expression. Additionally, the restricted T_FH_ differentiation caused by EZH2-deficiency could be rescued by forced Bcl6 expression, which expression was assured by TCF1 (25). Although other functional target loci cannot yet be excluded, our observation demonstrated that the elevated methylation level of *Tcf7* locus was associated with the increased expression of DNMTs in T_FH_ cells. Further investigations are needed to determine whether DNMT1 or DNMT3B could act alone, or they coordinately associated with methylation level at *Tcf7* locus.

It is still poorly understood what recruits DNMTs to regions of DNA loci that become methylated in T cells. In our study, the expression of DNMT1 and DNMT3B were elevated in T_FH_ cells with EZH2 deletion, and the T_FH_ lineage associated gene *Tcf7* appears to be regulated by DNA methylation (**Figure 3**, **Supplemental Figure 2**). These findings prompt us to hypothesize that transcriptional repressor UHRF1 is a possible binding partner of DNMTs. UHRF1 is a PHD domain protein defined as the cooperator of DNMT1 (36–38). The PHD domain has been reported to interact with H3K18ac, which is associated with more accessible chromatin states (39). Thus, UHRF1 may serve as a link between DNMT1 and H3K18ac. Meanwhile, the expression of *Kat2a* and *Kat2b* (acetyltransferases of H3K18ac) were much higher in T_FH_ cells than that in T_H_1 cells (data not shown). These facts suggest that H3K18ac might be associated with the expression of T_FH_ lineage associated genes, including *Tcf7*. We suggest that DNMT1 may be driven to the H3K18ac associated *Tcf7* locus by binding with UHRF1 for methylating the proper sites. However, it needs to be further disclosed.

In the scenario of acute viral infection, naive SMARTA cells differentiate into T_FH_ and T_H_1 cells (40–43). The bifurcation of T_FH_ and T_H_1 cells is mediated by Bcl6, and the expression of Bcl6 is ensured by TCF1. We observed that the methylation status at *Tcf7* locus was more heavily in T_H_1 cells than that in T_FH_ cells, while there was no detectable difference in the methylation status at *Bcl6* locus between T_FH_ and T_H_1 cells. Besides, the expression of DNMT1 and DNMT3B were comparable in T_FH_ and T_H_1 cells, and the DNMT3A expression was much higher in T_FH_ cells compared with that in T_H_1 cells (data not shown). These results indicated that *Tcf7* locus may also be the target locus of DNA demethyltransferase. Although the precise mechanisms about the additional modifications affect the methylation status at *Tcf7* locus are not clarified yet, our results suggest that the hypomethylation of *Tcf7* locus but not *Bcl6* locus was associated with the differentiation of T_FH_ cells.

In summary, this study reveals for the first time that EZH2 regulates the epigenetic modification of *Tcf7* during acute viral infection. EZH2 plays a crucial role in modulating the degree of DNA methylation at *Tcf7* locus and promoting the differentiation of T_FH_ cells. Since T_FH_ cells are critical in humoral immunity responses and development of autoimmune diseases, the dissection of EZH2 function may provide substantial therapeutic benefits for the treatment of viral infection and autoimmune diseases.

## Data availability statement

The original contributions presented in the study are included in the article/supplementary material. Further inquiries can be directed to the corresponding authors.

## Ethics statement

The animal study was reviewed and approved by Institutional Animal Care and Use Committee of the Third Military Medical University.

## Author contributions

YL, DL, ZW, LX, SL, XC, CW, LL, SY, FL, and XX performed the experiments. ZL, LH, JT, DY, JF, CC, and YZ provided reagents, materials and support. RH and LY designed the study, analyzed the data and wrote the paper with YL and ZW. RH and LY supervised the study. All authors contributed to the article and approved the submitted version.

## Funding

This work was supported by National Key Research and Development Program of China (2020YFA0804400), National Natural Science Foundation of China (81871248 to RH, 32000619 to ZW), China Postdoctoral Science Foundation (2020M673647 to ZW), Chongqing Special Postdoctoral Science Foundation (cstc2020jcyj-bshX0039 to ZW) and Program of HUST Academic Frontier Youth Team (2018QYTD10).

## Acknowledgments

We thank R. Ahmed (Emory University) for LCMV Armstrong virus and SMARTA transgenic mice. We thank the core facility center of Third Military Medical University for cell sorting.

## Conflict of interest

The authors declare that the research was conducted in the absence of any commercial or financial relationships that could be construed as a potential conflict of interest.

## Publisher’s note

All claims expressed in this article are solely those of the authors and do not necessarily represent those of their affiliated organizations, or those of the publisher, the editors and the reviewers. Any product that may be evaluated in this article, or claim that may be made by its manufacturer, is not guaranteed or endorsed by the publisher.
