# Supplementary material for: EZH2 restricts Tcf7 DNA methylation and promotes TFH differentiation during acute viral infection
Source: Front Immunol. 2022 Aug 15;13:942465. doi: 10.3389/fimmu.2022.942465 (PMC9420843; doi:10.3389/fimmu.2022.942465)
Supplement: Supplementary file 1 [file DataSheet_1.docx]

Supplementary Material

EZH2 restricts *Tcf7* DNA methylation and promotes T_FH_ differentiation during acute viral infection

## Supplementary Figure 1

**
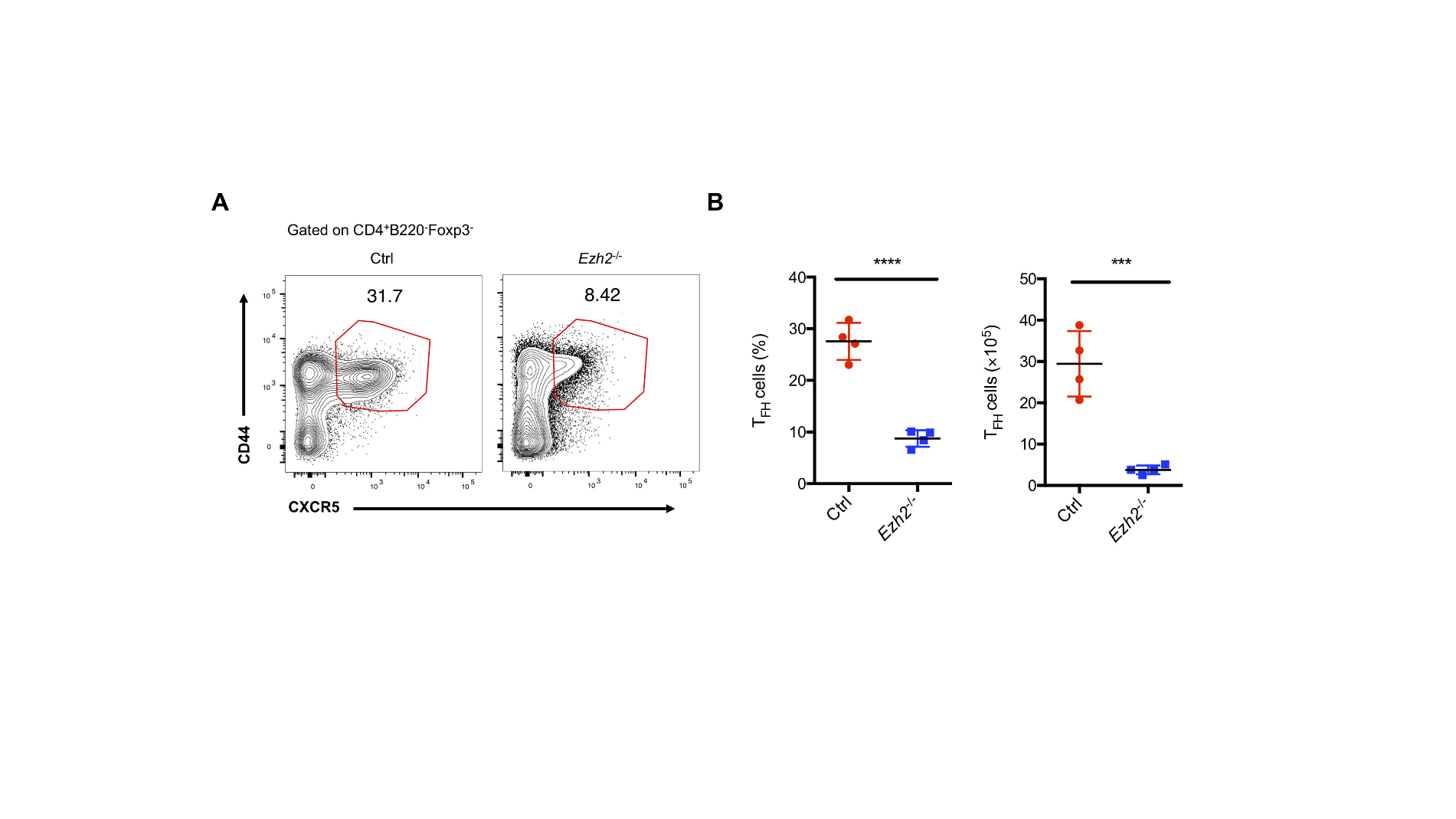
**

**Supplementary Figure 1.** (A, B) Quantification of frequency and number of T_FH_ cells from control and *Ezh2*^-/-^ mice at day 8 after acute viral infection. *P* value was calculated by unpaired two-tailed Student’s *t* test from triplicate experiments. Error bars indicate mean ± SEM, ****P* < 0.001, *****P* < 0.0001.

## Supplementary Figure 2


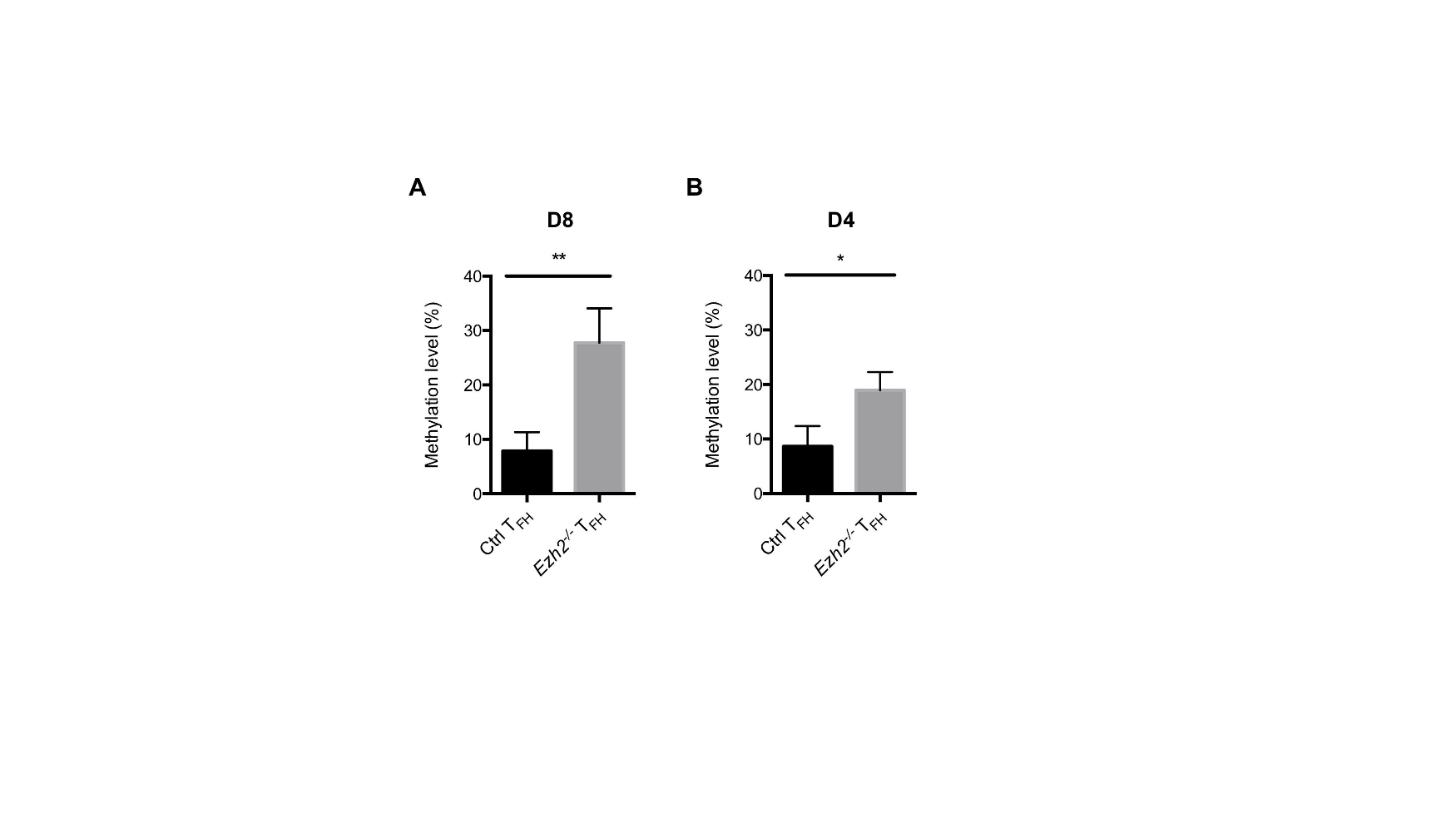


**Supplementary Figure 2**. Analysis of methylation level at the *Tcf7* locus in T_FH_ cells from control mice and *Ezh2*^-/-^ mice at day 8 (A) and day 4 (B) after LCMV Armstrong strain infection. *P* value was calculated by unpaired two-tailed Student’s *t* test from triplicate experiments. Error bars indicate mean ± SEM, **P* < 0.05, ***P* < 0.01.

## Supplementary Figure 3

**
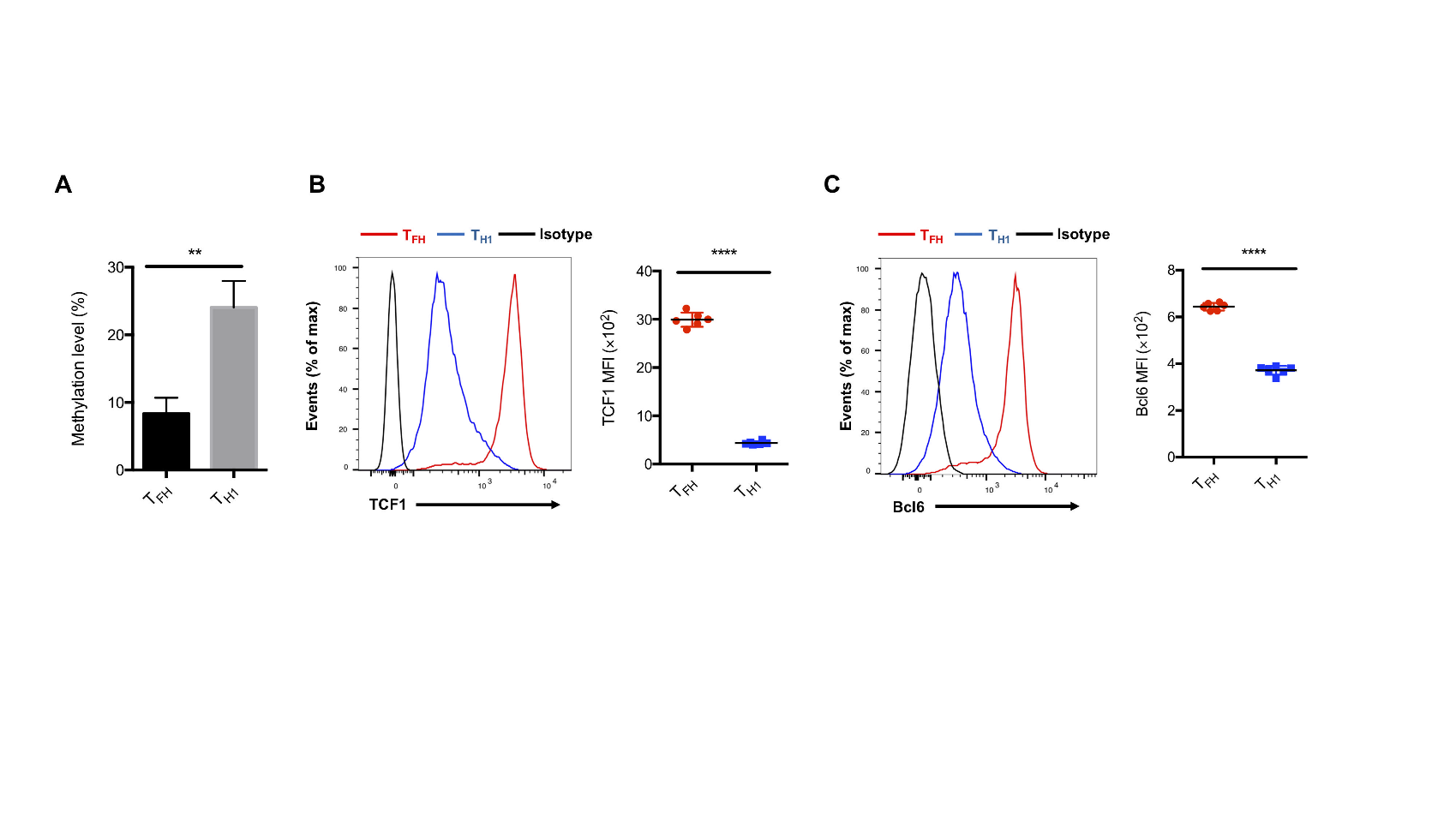
**

**Supplementary Figure 3**. (A) Analysis of methylation level at *Tcf7* locus in antigen specific T_FH_ cells and antigen specific T_H_1 cells at day 7 after LCMV Armstrong infection. Measurement of TCF1 (B) and Bcl6 (C) expression in antigen specific T_FH_ cells and antigen specific T_H_1 cells. *P* value was calculated by unpaired two-tailed Student’s *t* test. Error bars indicate mean ± SEM. ***P* < 0.01, *****P* < 0.0001.

## Supplementary Figure 4


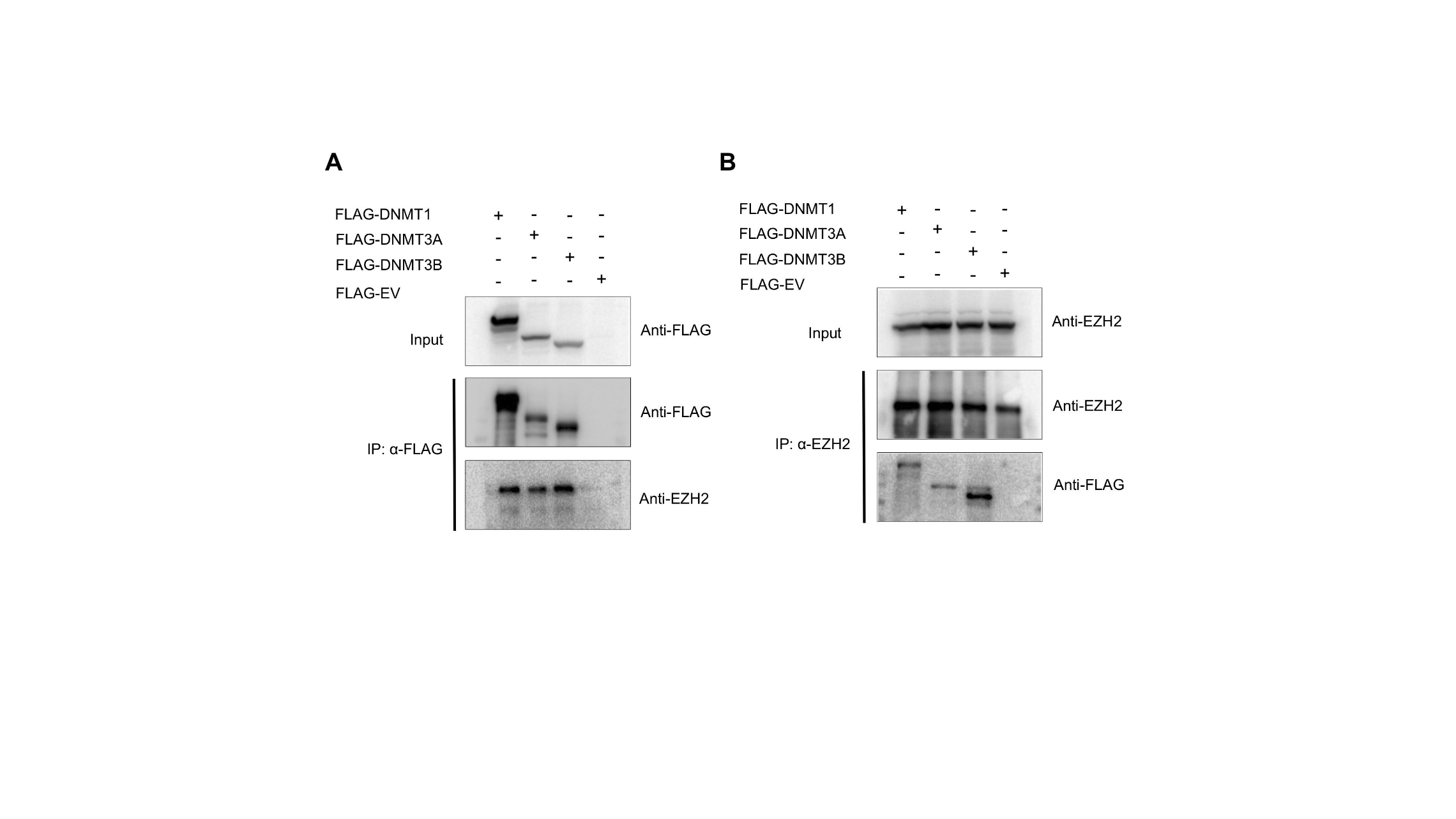


**Supplementary Figure 4**. The 293T cells were transiently infected with FLAG-tagged DNMT1, DNMT3A, DNMT3B plasmids. We conducted the lysates with co-immunoprecipitation followed by immunoblot analysis. (A) The cell lysates were immunoprecipitated with Anti-FLAG M2 Affinity Gel, followed by immunoblot analysis with anti-FLAG, and anti-EZH2 antibody. (B) Immunoprecipitation of extracts with purified EZH2 antibody, followed by immunoblot analysis with anti-EZH2, and anti-FLAG antibody.

**Supplementary Table 1**

| **For gene expression analysis** | | |
| --- | --- | --- |
| Gene name | 5'-3' | 3'-5' |
| *Tcf7* | caatctgctcatgccctacc | cttgcttctggctgatgtcc |
| *Bcl6* | agacgcacagtgacaaacca | agtgtgggtcttcaggttgg |
| *Prdm1* | agtgcaatgtctgtgccaag | ttgagattgcttgtgctgct |
| *Tet1* | tgctcatcctcaccagtctcttcc | gccgctcatcttccacctgaca |
| *Tet2* | tgtgagacggcggtgatggtaa | tctgagaacagcgacggttggt |
| *Tet3* | ctccttctccttcggctgttcct | catcctcattggtcacctggttctg |
| **For bisulfite sequencing** | | |
| Geneome location | 5'-3' | 3'-5' |
| *Tcf7 locus* | gaggtggaagaggtttatattgattt | ataacatctccttaacacccaacat |
| *Bcl6 locus* | aaggtyggatattaggtgattat | aaaaacttaaaaccaaaacatttaacaaa |
| *Prdm1 locus* | tgttttgtaggttaaagagggaa | cctactacctaccctatatttcaa |
| *Id3 locus* | gttaattgggtggaggtagtgtg | aaactcatccataccctcaaactt |
